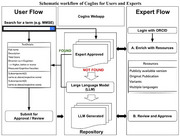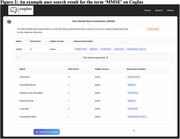# A Central, Community‐driven Repository for Neuropsychological Assessments in Alzheimer's and Dementia Research

**DOI:** 10.1002/alz70858_105375

**Published:** 2025-12-26

**Authors:** Bhargav Teja Nallapu, Laura A. Rabin, Richard B. Lipton, Ali Ezzati

**Affiliations:** ^1^ Technical University of Delft, Delft, Zuid‐Holland, Netherlands; ^2^ Albert Einstein College of Medicine, Bronx, NY, USA; ^3^ Brooklyn College of the City University of New York, Brooklyn, NY, USA; ^4^ The Graduate Center, CUNY, New York, NY, USA; ^5^ University of California, Irvine, Irvine, CA, USA

## Abstract

**Background:**

Comprehensive neuropsychological assessments are essential for characterizing cognitive deficits in Alzheimer's Disease and related dementias (ADRD), providing essential data for modeling disease progression and heterogeneity. However, the availability of multiple versions of similar tests, variations in language translations, and the existence of localized normative data make selecting and interpreting these assessments challenging for researchers, especially when working with demographically diverse individuals. Current resources, such as review articles and expert guidance, are limited in scope and accessibility, leaving gaps in knowledge and usability.

**Method:**

To address these gaps, we developed a centralized, open‐source repository of neuropsychological instruments that enables users to search for tests using various criteria, including name, geographic region, population of interest, and language. For each test, the repository provides comprehensive, curated information on its purpose, subcomponents, and cognitive domains assessed. The outcomes include metadata on source of the information, availability of local norms, language versions, and alternate test forms. For instruments not available in the repository, large language models (LLMs) generate preliminary summaries from existing literature, which can be submitted for expert review and refinement (Figure 1). The repository also supports crowdsourcing and community contributions to enrich the repository by incorporating information about original publications, translations, and test modifications.

**Result:**

The repository, named CogIns (Cognitive Instruments), is freely accessible at https://cogins.github.io and continues to expand daily. Details of several cognitive instruments are available with appropriate tags of AI Generated or Under Review (Figure 2). Preliminary user feedback highlights the tool's ability to streamline the knowledge organization related to the cognitive instruments and improve research accessibility, particularly for interdisciplinary researchers starting in the field of ADRD and those working with underrepresented populations.

**Conclusion:**

This repository represents a significant advance in democratizing access to neuropsychological assessment tools in ADRD research. It not only simplifies the process of identifying suitable tests but also establishes a scalable model forcommunity‐driven knowledge dissemination. Future updates aim to expand the repository's coverage, onboard experts to validate and curate the community‐generated information and introduce enhancements such as recommendations for relevant research, support for alternative languages, and access to publicly available test versions.